# Towards accurate monitoring of water content in woody tissue across tropical forests and other biomes

**DOI:** 10.1093/treephys/tpae076

**Published:** 2024-07-02

**Authors:** Lion R Martius, Maurizio Mencuccini, Paulo R L Bittencourt, Moisés Moraes Alves, Oliver Binks, Pablo Sanchez-Martinez, Antonio C L da Costa, Patrick Meir

**Affiliations:** School of GeoSciences, University of Edinburgh, King's Buildings, Alexander Crum Brown Rd, Edinburgh EH9 3FF, United Kingdom; CREAF, Campus UAB, Cerdanyola del Vallés 08193, Spain; ICREA, Barcelona 08193, Spain; Geography, College of Life and Environmental Sciences, University of Exeter, Amory Building, Exeter EX4 4RJ, United Kingdom; Instituto de Geociências, Universidade Federal do Pará, Belém, PA 66075-110, Brazil; CREAF, Campus UAB, Cerdanyola del Vallés 08193, Spain; School of GeoSciences, University of Edinburgh, King's Buildings, Alexander Crum Brown Rd, Edinburgh EH9 3FF, United Kingdom; Instituto de Geociências, Universidade Federal do Pará, Belém, PA 66075-110, Brazil; Museu Paraense Emílio Goeldi, Belém, PA 66040-170, Brazil; School of GeoSciences, University of Edinburgh, King's Buildings, Alexander Crum Brown Rd, Edinburgh EH9 3FF, United Kingdom

**Keywords:** dielectric permittivity, drought response, forest ecosystems, frequency domain reflectometry, hydraulic capacitance, palms, vegetation water content

## Abstract

Forest ecosystems face increasing drought exposure due to climate change, necessitating accurate measurements of vegetation water content to assess drought stress and tree mortality risks. Although Frequency Domain Reflectometry offers a viable method for monitoring stem water content by measuring dielectric permittivity, challenges arise from uncertainties in sensor calibration linked to wood properties and species variability, impeding its wider usage. We sampled tropical forest trees and palms in eastern Amazônia to evaluate how sensor output differences are controlled by wood density, temperature and taxonomic identity. Three individuals per species were felled and cut into segments within a diverse dataset comprising five dicotyledonous tree and three monocotyledonous palm species on a wide range of wood densities. Water content was estimated gravimetrically for each segment using a temporally explicit wet-up/dry-down approach and the relationship with the dielectric permittivity was examined. Woody tissue density had no significant impact on the calibration, but species identity and temperature significantly affected sensor readings. The temperature artefact was quantitatively important at large temperature differences, which may have led to significant bias of daily and seasonal water content dynamics in previous studies. We established the first tropical tree and palm calibration equation which performed well for estimating water content. Notably, we demonstrated that the sensitivity remained consistent across species, enabling the creation of a simplified one-slope calibration for accurate, species-independent measurements of relative water content. Our one-slope calibration serves as a general, species-independent standard calibration for assessing relative water content in woody tissue, offering a valuable tool for quantifying drought responses and stress in trees and forest ecosystems.

## Introduction

The need for accurately quantifying vegetation water content (WC) has never been greater, as climate change exposes ecosystems to more extreme, more frequent and longer droughts (Dai 2013, Gentine et al. 2019, Hammond et al. 2022, IPCC 2022). The resistance of plants to water deficits is commonly assessed using mortality risk metrics, which in the past have primarily focused on describing water flows such as embolism-induced alterations of conductance and stomatal behaviour (Eller et al. 2020, Sanchez-Martinez et al. 2023). A focus on measuring water pools, particularly stem WC, has developed in recent years with the potential to greatly improve studies of drought-induced mortality risks, vegetation water use and storage dynamics over time and space (Saatchi et al. 2013, Martinez-Vilalta et al. 2019, Konings et al. 2021, Chaparro et al. 2024). Stem water storage forms a crucial buffer against hydraulic impairment caused by daily transpiration demand, especially when soil water availability is limited (Goldstein et al. 1998, Meinzer et al. 2009, Hao et al. 2013, Salomón et al. 2017, Ziemińska et al. 2020). Despite the importance of internally stored water in regulating whole-plant water status, high temporal resolution data on diurnal and seasonal dynamics of tree WC are sparse, especially in the tropics (Matheny et al. 2017, He et al. 2021). Vegetation volumetric (VWC, m^3^_water_ m^−3^_wood_) and relative water content of woody tissue are still predominantly measured gravimetrically, a destructive method for determining WC that cannot easily resolve temporal differences at high resolution (Ziemińska et al. 2020, Pereira Dias and Marenco 2021). Plant WC sensors provide an effective method for studying tree water relations; however, significant uncertainty remains on the theory of their use, calibration and validation. These constraints have tended to limit wider usage.

Frequency domain reflectometry (FDR) is a technology used to measure WC of porous materials and is widely used in soil science to monitor soil WC. FDR sensors estimate the dielectric permittivity (ε) around a wave guide, that is, the capacity of the medium surrounding the wave guide to hold electric charge. At a range of frequencies (10^6^ to 10^9^ Hz), ε becomes almost only sensitive to WC (Davis and Annan 1977). As a consequence, the ε of porous materials such as woody tissues or soils at those frequencies is proportional to VWC (Topp et al. 1980). Capacitance sensors have gained increasing attention for measuring vegetation WC in vivo (Hao et al. 2013, Matheny et al. 2017, Salomón et al. 2020, He et al. 2021). However, the calibrations derived for soils were not suitable for accurate WC estimations in woody tissue. In addition, the calibration equations derived for these materials varied across species and studies hindering the establishment of a universally applicable approach to estimate plant WC by measuring ε in woody tissue (Holbrook et al. 1992, Matheny et al. 2017, He et al. 2021). As a result, species-specific calibrations have been recommended (Matheny et al. 2017). Although this approach has proven effective in ecosystems with a low tree species diversity, the high diversity of sub/tropical forests make species-specific calibrations impractical.

As the FDR signal in soils is influenced by the porosity of the medium (Nasta et al. 2024), it had been previously proposed that the signal in woody tissue could be predicted by wood density and porosity as well. In regards to the latter, previous studies have highlighted that species-specific calibration curves in trees that exhibit contrasting porosity types, such as ring, semi-ring or diffuse porosity, or within monocotyledonous palms, differ significantly (Steppe and Lemeur 2007, McCulloh et al. 2010, Matheny et al. 2015). However, the effect of wood density on the FDR signal remains yet unexplored (He et al. 2021).

We also note the need to test for temperature sensitivity of FDR sensors and its potential to bias WC estimates when analysing diurnal and seasonal trunk water storage dynamics. The dielectric constant of many materials such as water is inversely proportional to temperature, meaning it decreases with increasing temperature. This is caused by the reduced polarization capability of molecules, with increasing temperature and thus increased thermal motion. However, for reliable measurements, it is important that the temperature sensitivity is insignificant in relation to the signal of interest.

The aim of this study was to calibrate electromagnetic sensors with underlying FDR technology across a wide range of wood density (WD) within diffuse-porous tropical hardwood species and to explore the WD and temperature effect on the calibration curves. To test the importance of both WD and porosity, we included anatomically different monocotyledonous palms. Palms do not display secondary growth and have tracheids within vascular bundles and vessel elements scattered around their stem tissue, mainly surrounded by parenchyma. We thus hypothesize that the calibration for arborescent palms would differ from that for dicotyledonous trees, while WD would be the principal variable determining the calibration curve for trees of the same internal architecture and sapwood porosity type. We further hypothesize that the effects of species and WD on calibration curves act on the intercept rather than slope of the relationship between sensor output and wood WC, as the intercept can be influenced by the density and structural differences of the wood, whereas the change in permittivity should be mainly driven by changes in WC independently of woody tissue density and structure. Moreover, we suggest that temperature significantly affects the sensor reading; however, the temperature effects in tropical subcanopies are negligibly small, in contrast to highly seasonal biomes.

Therefore, our main research questions of this study are: (i) is there a difference in the calibration signal between monocotyledonous palms and tropical dicotyledonous trees? (ii) Does a single calibration exist for tropical dicot trees independent of species identity, potentially adjusted for woody tissue density? (iii) How large is the bias introduced by temperature fluctuations and what are the implications for quantifying temporal trunk water storage dynamics? A key goal of this study is to understand to what extent external and internal factors influence FDR sensor calibration for woody tissue to overcome the limitations for the establishment of a general standard calibration for estimating vegetation WC using dielectric permittivity ε.

## Materials and methods

### Study site

The study was conducted in an Amazonian lowland tropical rainforest situated in the Caxiuanã National Forest (Floresta Nacional de Caxiuanã), state of Pará, in northern Brazil (1°43′S, 51°27′W). The calibration was performed during two field campaigns in November–December 2022 and February–March 2023 at the biological field station Estação Científica Ferreira Penna. The site is designated as an old-growth terra firme forest and is located within Tocantins–Araguaia–Maranhão moist forests ecoregion, the most easterly in the Amazon basin. The seasonally dry evergreen forest receives an annual precipitation of between 2000 and 2500 mm, with monthly rainfall dropping below <100 mm month^−1^ during the dry season (June—November). The equatorial climate results in steady temperatures throughout the year with a mean below-canopy temperature of 26 °C (Meir et al. 2018).

### Species sampling and wood characteristics

The calibration of FDR sensors was performed on five species of dicotyledonous tropical forest trees and three monocotyledonous palm species (Table 1). Three individuals per species were cut into segments (see `Calibration of capacitive Teros-12 sensor for tropical woody tissue'). Species were selected based on local abundance and were chosen to cover a wide range of WD. Wood density was later re-measured for all segments and species during this study. The dicotyledons from this study exhibited diffuse-porous xylem and palms were added to the dataset, with a contrasting wood anatomy.

**Table 1:** Sampled species and their wood characteristics divided into dicotyledonous trees and monocotyledonous palms in ascending order of wood density (WD).

Species		WD	Porosity
*Jacaranda copaia*	D.Don	0.29 ± 0.02	Diffuse-porous
*Protium tenuifolium*	Engl*.*	0.54 ± 0.03	Diffuse-porous
*Licania octandra*	Kuntze	0.69 ± 0.05	Diffuse-porous
*Vouacapoua americana*	Aubl.	0.82 ± 0.05	Diffuse-porous
*Manilkara bidentata*	A.Chev.	0.84 ± 0.04	Diffuse-porous
*Euterpe oleracea*	Mart.	0.22 ± 0.04	Monocotyledonous palm
*Oenocarpus distichus*	Mart.	0.29 ± 0.07	Monocotyledonous palm
*Astrocaryum vulgare*	Mart.	0.63 ± 0.21	Monocotyledonous palm

Values for WD were measured during this study for each sample. Values are given as species-specific means (± SD) in g cm^−3^.

### Calibration of capacitive Teros-12 sensor for tropical woody tissue

Three-pin Teros12 moisture sensors with underlying FDR technology (Meter Group, Pullman, WA, USA) were used for the calibration of the different tropical hardwood species and palms. The initial probe of 5.5 cm was shortened to 3 cm length, making sure that the majority of the wave guides were situated within the sapwood. This was supported by previously observed allometric relationships between sapwood area and diameter at breast height (DBH) found in Amazonian trees, with an average of 3.5 cm sapwood depth for trees with an average DBH of 19.2 cm (Aparecido et al. 2019). Frequency domain reflectometry sensors measure the ε of the medium. There is a known physical relationship between ε and water concentration (Davis and Annan 1977, Topp et al. 1980). Electrical permittivity was calculated from the measured raw values using the transformation equation provided by the manufacturer’s manual for Teros11/12 (SI II). Due to the nonlinear nature of the relationship between ε and WC, square root transformations are commonly employed to fit simpler linear calibration models (He et al. 2021).

For each tree species, three individuals were identified and felled (stem diameter: 12 to 20 cm) to be cut into 8 to 15 segments (depending on practical stem length), each 20 cm in length (total *n* = 262; reflecting total number of measuring points, as each wood segment was measured only once). The segments were harvested starting from the base of the trunk with increasing height. Immediately after felling and cutting, the segments were wrapped in cling film and transported to the nearby field laboratory. Each wood segment was then predrilled with a custom-made drill-guide for sensor placement and readings of the fresh wood WC, temperature and the raw sensor output were taken. This helped us understand the variability of FDR sensor outputs from fresh wood samples across species. The sensor–wood interface was not sealed as many sensors had to be reused. The 3-mm drill bit was slightly thinner than the sensor needles (3.175 mm), assuring close contact between the sensor and woody tissue. The drill bits were marked at 30-mm length, so that sensor needle equalled the length of the drill holes. After the FDR measurement, the sensor was removed from the segment and the fresh mass (m_f_) was measured and the fresh volume (V_f_) was determined using the Archimedes principle. Subsequently, the wood segments from each individual tree were divided into two groups for re- or dehydration, with ~1/3 of the segments being transferred into a water bucket for wetting-up and 2/3 into a field oven for drying-down. For each individual, segments were measured at different times spent within the above-mentioned treatment. One rehydrating segment was taken out of the water bucket and measured with the FDR sensor every second day, after any excess water on the outer surface of each segment had been carefully removed, using paper tissue. For the dehydration segments, the temperature of the drying oven was set to 70 °C. Depending on the dehydration rate, a segment was taken out at either daily or twice-daily intervals for FDR sensor measurements. Prior to these measurements, segments were wrapped in plastic film and left to equilibrate for 12 h to avoid bias caused by uneven drying in the oven. After taking the FDR measurement, the sensor was removed again and the weight of each segment was recorded before the de-/rehydrating segment was transferred back into the oven for full desiccation. The state of full desiccation was assumed after stabilization of the loss of mass from the wood over the course of 2 days. Segments usually took between 2 and 3 weeks to dry down fully. The dry mass of each segment was measured and the gravimetric VWC was determined as the ratio of the volume of stored water (converted from mass) to the unit of fresh volume of the wood, $\frac{({m}_f - {m}_d)\ }{V_f\ }$ (m^3^_water_ m^−3^_wood_), where ${m}_f$ and ${m}_d$​ are the fresh and dry masses of the wood, respectively. Following the calculations for gravimetric VWC, the values for the oven dry mass (m_d_) and fresh volume (V_f_) were used to calculate WD, defined as the ratio of $\frac{m_d}{V_f}$ (g cm^−3^). Each wood segment was measured only once; however, there were multiple replicates (segments) taken for each individual tree nested within three replicate trees for each species.

### Testing temperature sensitivity on FDR sensor output

To quantify the effect of temperature on the FDR sensor output, the two following experiments were designed—the ‘water bucket’ and ‘wood block’ experiment. For the former, three shortened Teros12 sensors were placed in a water bucket inside a cool box filled with water from the fridge and left to warm up naturally in the shade over the course of 2 days, while temperature and FDR WC data were logged at 5-min intervals (temperature range: 15 to 27 °C). Sensors were placed > 10 cm away from each other to avoid signal interferences. In parallel, for the wood block experiment, one Teros12 sensor was inserted into a block of wood and wrapped in cling film to keep the WC constant. The wood block was placed into a cool box where the T slowly increased to ambient temperatures (temperature range: 16 to 27.5 °C). Data were logged with a ZL6 logger (Meter Group, Pullman, WA, USA). In addition to the calibration study, we readily installed a shortened FDR sensor in a living tree (*Manilkara bidentata* A.Chev.) and logged VWC, raw and temperature data in 15-min intervals for several months. This allowed us to discuss the significance of temperature corrections for water storage dynamics in a lowland tropical forest tree. Additionally, an installation and data processing protocol has been developed for the use and calibration of electromagnetic sensors in the field and published on GitHub (SI III).

### Statistical analysis and model comparison

Linear mixed-effect models were used to quantify the fixed effects of electrical permittivity and wood density on gravimetric VWC (Bates et al. 2015, Kuznetsova et al. 2017, Bartoń 2023). Each wood segment represented one datapoint; however, we conducted multiple measurements of different segments within individuals nested within species. Hence, these terms were set as random variables. Up to 10 sensors were randomly deployed within the same individual and species to mitigate the influence of sensor-specific variations on species-specific disparities.

Linear models were used for estimating the temperature effect on the FDR signal. Linear models were also used to develop a species-specific calibration equation suitable for trees in the tropics (Tropical Tree Calibration—TTC).


$$ {\theta}_{stem}=m\ \sqrt{\varepsilon_{stem}}-b $$

Furthermore, a linear model using species ID as an interaction was used to test for an interaction effect between species, which was compared to a main effects linear model (SI VI Table 2). We evaluated the feasibility of a one-slope calibration (OSC) for measuring relative changes independent of species identity by removing intercept effects by applying first-order differencing to the species-specific data for $\sqrt{\varepsilon }$ and gravimetric VWC. The differenced points were calculated by taking the difference between consecutive observations within each species-sample combination. Each differenced point is relative to the preceding time point. We then tested if we could retrieve the same accuracy as for the mixed effect models accounting for the species effect.

Model accuracy was quantified using the root mean square error (RMSE), an estimate of the random error term. Additionally, the mean absolute error (MAE) and relative absolute error (RAE) were reported as systematic bias estimates. The coefficient of determination (*R^2^*) was used to quantify the model goodness-of-fit. For mixed effect models, both the marginal (${R}_m^2$) and the conditional $\left({R}_c^2\right)$ coefficients of determination were reported to quantify how the model improved by including random variables. We tested the significance of the mixed model effects using a likelihood ratio test (LRT). All statistical analyses were conducted in R (RStudio Team 2020).

## Results

### Drivers of variability in FDR calibration

**Figure 1:**
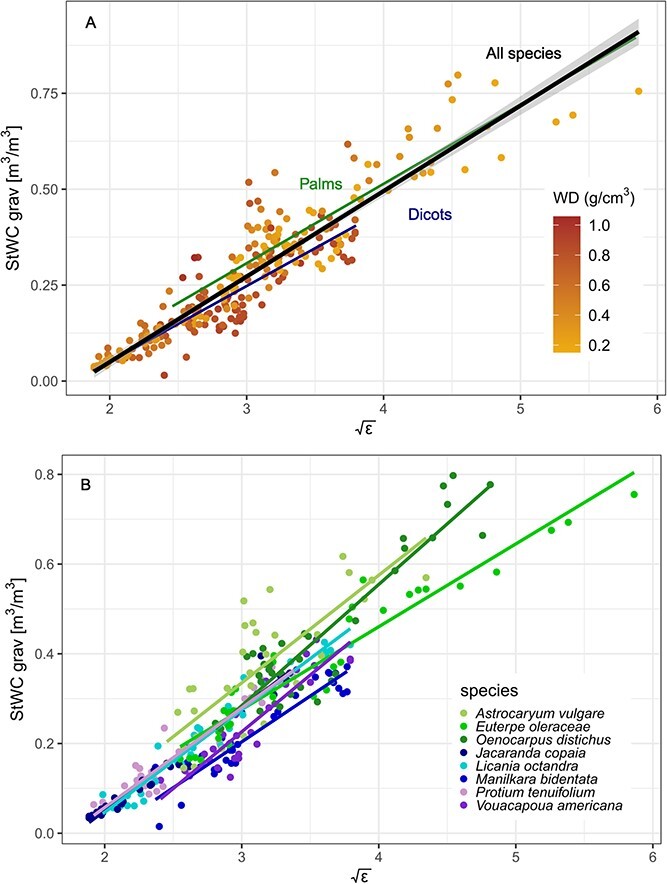
Factors affecting the calibration curve for frequency-domain reflectometry sensors. In contrast to previous findings, palms are grouped very closely to dicotyledonous tree species. Furthermore, while (A) wood density had no significant effect on the calibration curve, (B) random species effects accounted for more variation than WD. To improve readability, the graph has been simplified by aggregating data at the species level, omitting individual trees.

With a total of 262 wood segments from five dicotyledonous trees and the three monocotyledonous arborescent palm species (*n* = 3 individuals per species), this study presents the largest single source calibration dataset available to date for estimating wood WC by measuring dielectric permittivity (ε) using FDR technology in woody tissue. Contrary to our expectations, the calibration curve fitted for monocotyledonous palms was highly similar to the dicotyledonous tree species (Fig. 1). In addition to taxonomic variation, the dataset contained a wide range of values for WD covering an important proportion of the range of WD found within natural forest ecosystems. Wood density ranged from species means for palms of 0.22 g cm^−3^ (*Euterpe oleracea*), 0.63 g cm^−3^ (*Astrocaryum vulgare*), 0.29 g cm^−3^ (*Jacaranda copaia*) and to 0.84 g cm^−3^ (*M. bidentata*) in dicot trees (Table 1). Our linear mixed effect model showed that the fixed predictor of species mean WD had no significant effect on the calibration fit and it was thus excluded from the model (Fig. 1A). The majority of the variation in the model was explained by $\sqrt{\varepsilon }$ alone (${R}_m^2$ = 0.84, *t*_(259)_ = 38.3, *P* < 0.001). However, results suggest that a significant proportion of the variation influencing the calibration was attributed to the random intercept effects of species ID (LRT = 13.1, *P* < 0.001) and individuals nested within species (LRT = 10.6, *P* < 0.001). Together these effects improved model performance by < 10% (${R}_c^2$ = 0.91, Fig. 1B).

### A tropical tree calibration

Despite species-specific differences in the intercept, all plants from this study were found to be closely grouped, including arborescent palms. Hence, we established a simple linear calibration equation for tropical trees (TC) to estimate WC (θ) including palms and based on ε only:


$$ {\theta}_{stem}=0.2227\sqrt{\varepsilon_{stem}}-0.396 $$

The TTC performed well (${R}^2$= 0.84); however, the calibration accuracy was lower than the 3% stated as the RMSE by the manufacturer (MAE = 0.047, RMSE = 0.064 and RAE = 0.36).

#### OSC for species independent measurements of relative WC

The ANOVA results from a linear model, including $\sqrt{\varepsilon }$ and species ID as a fixed effect, indicated that there was a significant interaction effect between species and $\sqrt{\varepsilon }$ (*F*_(7, 245)_ = 4.07, *P* < 0.001). However, the overwhelming majority of the variation was explained by $\sqrt{\varepsilon }$ (*F*_(1, 245)_ = 2397.44, *P* < 0.001) and the species-specific intercepts (*F*_(7, 245)_ = 23.9 *P* < 0.001) within this model, rather than the slope. Furthermore, there was no significant improvement in the model goodness-of-fit between the interaction effect model and the main effects model alone, further supporting the idea that differences in the intercept explain the majority of the variation and not the slope (SI VI Table 2).

**Figure 2:**
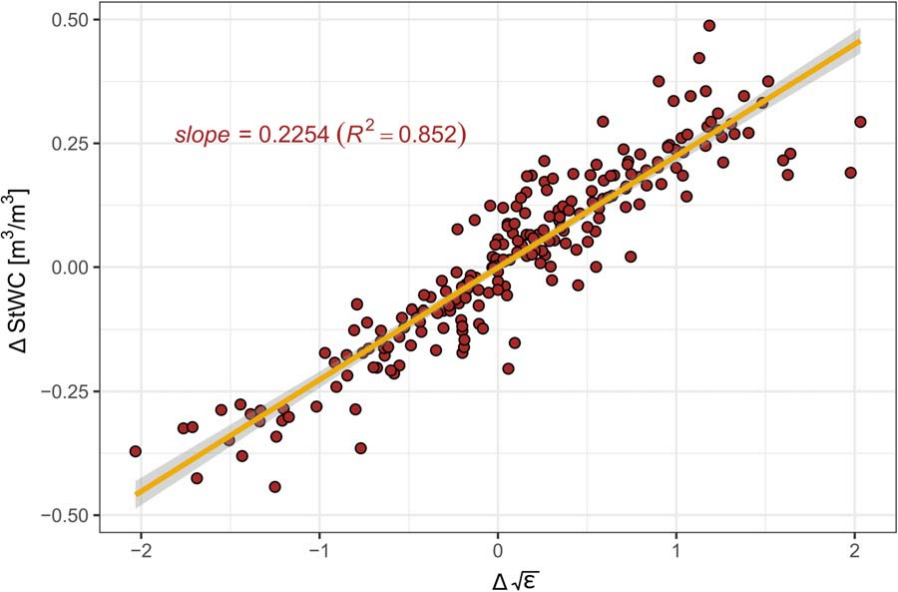
As species-specific differences were driven by intercept differences in the TTC, first-order differencing was applied to model the relative changes between StWC and $\mathrm{\varepsilon}$. This led to the development of an OSC, which disregards species-specific intercept changes and focuses on the relative changes**.**

To take advantage of the consistent slope, we applied first-order differencing within each species to establish a novel calibration based on one slope (OSC, *s* = 0.2254, 95% CI (0.2130, 0.2379)). This re-analysis removed the variability in species-driven intercepts by modelling how the change in StWC relates to the change in $\sqrt{\varepsilon }$ (${R}^2$= 0.852, MAE = 0.05, RMSE = 0.07 and RAE = 0.34, Fig. 2).

Our species-dependent calibration, the TTC, enables the accurate estimation of absolute WC values for the species sampled within this study. However, when installing the sensors in unknown trees or palms, its accuracy can be negatively affected by unknown intercepts. Our analysis indicated that the species-independent OSC model enables the measurement of relative changes in WC across unknown species while retrieving a similar accuracy as for a model accounting for species differences.

### Temperature effects on FDR sensor signal

**Figure 3:**
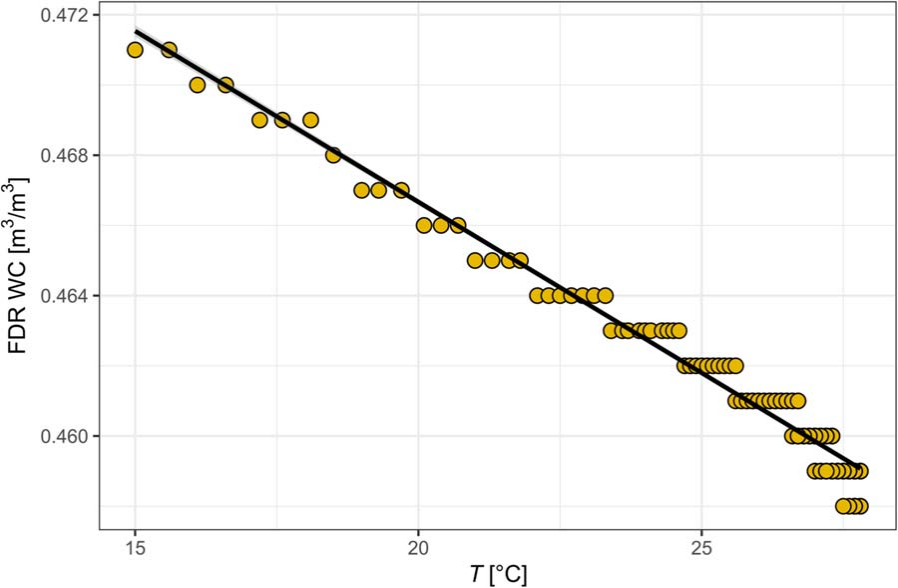
Linear model result of the temperature effect experiment conducted with uncalibrated Teros12 sensors in a water bucket. With increasing temperature, the dielectric permittivity decreases, affecting the FDR sensor reading.

The results from the water bucket test revealed that there was a significant and quantitatively important negative relationship between the FDR signal and temperature, with an estimated slope coefficient of −0.000974 m^3^m^−3^ °C^−1^ (${R}^2$= 0.96, *t*_(769)_ = −137, *P* < 0.001, Fig. 3), 95% CI (−0.000988, −0.000960). This equally refers to an effect on $\sqrt{\varepsilon }$ of −0.0145 °C^−1^ (${R}^2$= 0.96, *t*_(769)_ = −140.5, *P* < 0.001). Additionally, the wood block experiment also displayed a highly significant negative relationship between the FDR signal and temperature. Here, the slope coefficient was very similar at −0.000950 m^3^ m^−3^ °C^−1^ (${R}^2$= 0.53, *t*_(228)_ = −16, *P* < 0.001) and not significantly different from the water bucket experiment (SI IV Fig. 1)*.* Despite no differences in slope, the wood block experiment model performed less well due to condensation formation in a highly humid atmosphere, as the sensors were not sealed. Thus, the coefficient from the water bucket tests was used only.

**Figure 4:**
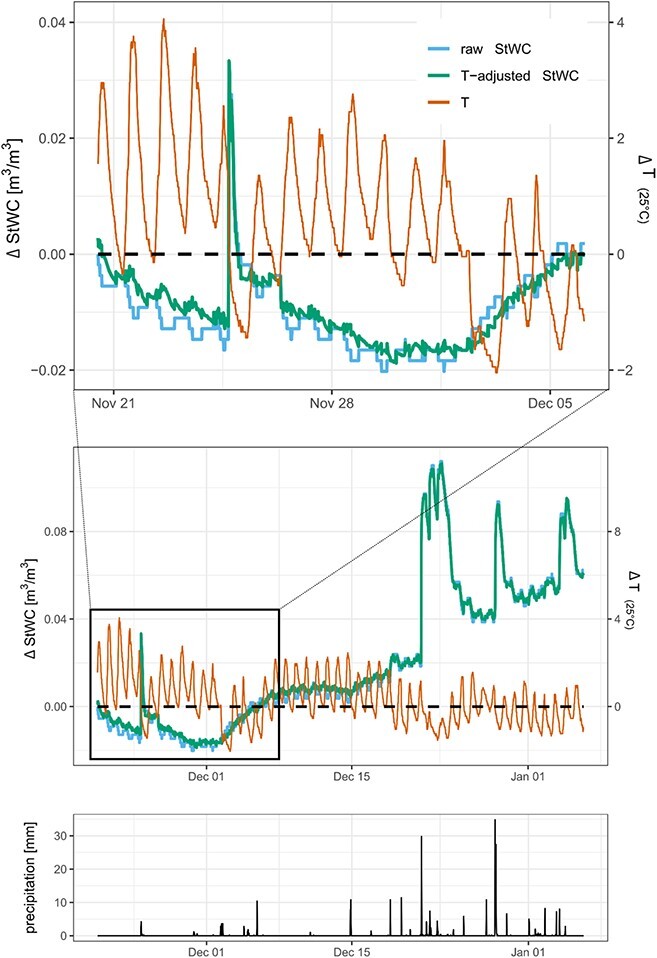
Field data from *Manilkara bidentata* in Caxiuanã showing the unadjusted ΔStWC and the temperature-adjusted ΔStWC. The ΔT relative to the mean (25 °C) is given on the secondary *y*-axis. While larger daily fluctuations of T in the dry season (upper panel) are mainly driving artefactual diurnal WC dynamics, a true diurnal WC dynamic exists in the wet season starting end of December (middle panel), even after accounting for temperature effects. Large changes in StWC coincide well with precipitation events at the start of the wet season (lower panel). Due to low ΔT in the tropics, seasonal data are relatively unaffected by T (middle panel). However, care must be taken when analysing diurnal patterns (upper panel), especially in the dry season. Disregarding the temperature effect leads to false physiological and ecological conclusions. Care must be taken when interpreting figure parts (upper panel) and (middle panel), as the axes are differently scaled.

At our study site in Caxiuanã, the below-canopy mean temperature difference between wet and dry season is <1 °C, whereas the daily mean temperature difference is more than 3 °C, with a maximum daily difference <5 °C (Fig. 4). If we assumed the T difference between day and night to be 5 °C, this would lead to a bias of 4.87 kg m^−3^. For larger temperature differences (ΔT), which occur in subtropical, temperate and boreal regions, the introduced bias from a ΔT of 10 °C, 20 °C and 30 °C, the artefactual changes in WC would be 9.74, 19.48 and 29.22 kg m^−3^, respectively.

## Discussion

### Drivers of inter-species differences in calibration

One of the underlying hypotheses of this study was that calibration differences would be explained by the density of the woody tissue. We sampled across a wide range (0.22 to 0.84 g cm^−3^) of wood densities commonly found in natural forest ecosystems, but our data did not show any significant or quantitatively important impact of WD. Additionally, we included three species of the anatomically different group of monocotyledonous palms, which have previously been reported to significantly differ in their dielectric response (Holbrook et al. 1992, He et al. 2021). However, we could not find any agreement with previously reported differences from palms of this study. We thus conclude that palms are in fact closely grouped to dicotyledonous trees and should not be considered differently. We suggest that our calibration equation provides a good basis for the collection of WC data on the physiologically and ecologically understudied group of palms (Aparecido et al. 2015, Emilio et al. 2019, Muscarella et al. 2020, Cooper et al. 2024). Field data on stem WC show substantial differences between palms and dicots, with the former containing up to twice as much volumetric water compared with dicots within their stems, underlying their importance in ecological dynamics within forests.

As WD did not explain calibration differences, it is likely other physico-chemical properties and the structural composition of the measured media may explain how the FDR signal responses differ among plant materials. In woody tissue, WC reductions are sensed because of: (i) changes in the position of the water meniscus inside the fibres; (ii) embolization of conduits and fibres; and (iii) decrease in stem volume leading to increases in dry mass fraction (the proportion of the mass of a plant which is not water). Stem shrinkage does lead to increases in cell wall fraction (the proportion of a plant’s volume occupied by cell walls). However, unless there is a significant change of the fibre volume, the FDR signal should be influenced mainly by air volume or water concentration changes, rather than WD (Gartner et al. 2004, Knipfer et al. 2019). This can be explained by the low and constant ε of lignin (D’Alvia et al. 2022). Furthermore, if the degree of embolism remains low and stem volume changes are not too large, it is possible that differences in wood porosity would have no significant impact on the calibration curves, a prediction supported by the close grouping of arborescent palms with diffuse-porous dicotyledonous trees in our data (Fig. 1). However, it is important to note that we found a small intercept difference between palms and the dicots and an even larger intercept difference between tropical and temperate trees from a previous meta-analysis (He et al. 2021). Wood anatomy of the latter differs significantly displaying vessel width differences in accordance to pronounced seasonality (Tyree and Ewers 1991).

### One slope perspective

**Figure 5:**
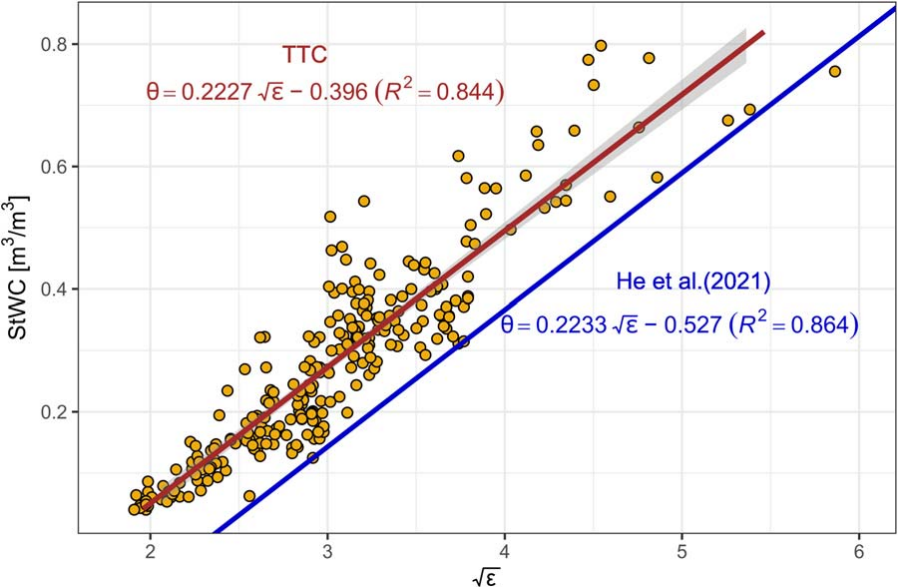
A single calibration (TTC) was established for the relationship between $\mathrm{\varepsilon}$ and StWC for tropical trees, including arborescent palms from this study. Interestingly, the slope (s = 0.223) established here was indistinguishable from the temperate tree model by He et al. (2021) and only the intercept was found to be different.

Apparent differences in the calibration curves previously found across species and studies led to the conclusion that species-specific calibrations are necessary. This significantly restricted the wider use of FDR technology to measure WC in trees, especially in biodiverse ecosystems. Thus far, only one large scale study exist that pools together large calibration datasets of FDR/TDR sensors, which aimed to establish a generic relationship between the dielectric permittivity and WC in woody tissue (He et al. 2021). We provide here the second large scale study, conducted in tropical trees and palms. We note that the slope found here for tropical woody tissue was indistinguishable from the slope estimated in a meta-analysis comprising data from several studies, including 12 tree species from boreal, temperate and Mediterranean regions and using different methodologies (*s*_(TTC)_ = 0.2227, 95% confidence interval (CI) (0.2111, 0.2344), *s*_(__He__et al.__(2021)__)_ = 0.2233; Fig. 5). Although our study only used one sensor type (FDR, modified Teros12), the slope remains consistent across different sensor types (TDR) and waveguide lengths (10, 12.5, 13, 15 cm), wood densities, porosity and taxonomic units when compared with the meta-analysis (He et al. 2021). We thus argue that this slope remains consistent in woody tissue in general. However, we are also aware that different slopes have been found in previous studies (Holbrook et al. 1992, Hao et al. 2013, Matheny et al. 2017, Salomón et al. 2020). Although we believe that these studies have been well designed, we argue that the calibration fit is highly sensitive to low sampling numbers and sampling range. Low sampling numbers make the calibration highly sensitive to introduced bias during the calibration. Measurement bias is introduced easily, despite careful study designs, as woody tissue is non-homogenous, leading to potential air gaps between the sensor and tissue. Other bias could come from not considering temperature differences during the calibration. Unprecise cutting of needle rods and thus sensor-specific differences can also significantly affect the calibration fit. Since we re-used sensors within the same individual at a random fashion during our calibration study, we tried to omit sensor bias; however, we cannot fully exclude these being present in our data. Thus, we believe that a strength of our study lies in a (i) large sampling number and repetitions across species and wood densities, (ii) wide sampling range from very dry to very wet wood and (iii) changes of sensors IDs within one individual, thus omitting errors introduced by sensor-specific variability caused by uneven cutting. Only after pooling together large datasets can the measurement bias be smoothed out. Hence, the slope found within our study is identical to the meta-analysis, despite vast differences in study design and methodology. This further highlights the complications associated with accurate species-specific calibrations and we hope to have removed some uncertainties to make the use of electromagnetic sensors in woody tissue widely applicable. We further propose how species-specific intercepts can be assessed in the field for the collection of absolute WC, which, however, as of yet lacks field data to support the idea (SI V).

Without the need to conduct calibrations, these low-cost sensors can be readily installed in stems, changing the quantity and quality of vegetation WC data that can be collected to assess tree water storage dynamics, which becomes highly relevant with accelerating climate change. Given the growing interest in monitoring vegetation WC through remote sensing technologies, FDR technology presents a promising method for ground-truthing the estimate of vegetation permittivity, which is required in the interpretation of microwave remote sensing data (Saatchi and Moghaddam 2000, Saatchi et al. 2013, Konings and Gentine 2017, Konings et al. 2021, Chaparro et al. 2024).

### Ecological and physiological consequences of temperature effect bias

The temperature-change experiment demonstrated the importance of incorporating temperature (T) sensitivity of these sensors into the calibration of FDR sensors for use in woody tissue. The results from this study are in accordance with previous work conducted in loamy soils using electromagnetic sensors (Or and Wraith 1999, Fares and Polyakov 2006, Nasta et al. 2024). Although the water-bucket experiment is relatively free of noise, the wood block experiment exhibits larger variation (lower *R^2^*, while maintaining the same slope). We were unable to air seal the sensors in the wood block experiment and conclude that the highly humid and warm atmosphere has led to condensation and increases in real WC. Temperature-induced variability in WC impedes separating the effect of T from WC changes in woody tissue. Thus, future studies should re-evaluate this thermal effect in different wood types, as a beneficial addition to the data presented here. Many such sensors are designed with a built-in thermistor to measure T inside the medium. In tropical understorey environments, variability in T is likely to have a negligible effect on the measurements at seasonal time scales, but may become important diurnally, especially if sensors are installed in highly exposed locations experiencing large T variation (e.g., in the crown or near canopy gaps, Fig. 4). Hence, the effect of T on the sensor’s signal as quantified earlier is likely to be considerably smaller in the wet tropics than in subtropical, temperate or boreal regions which experience large fluctuations in temperature diurnally and especially seasonally. Previous attempts in properly separating the temperature bias from thermal induced water storage dynamics have proven difficult, which was also the case in our wood block experiment. Thus, caution must be taken when applying temperature corrections. In fact, future studies should evaluate this effect across different wood types and a larger sampling range to improve the accuracy of the temperature correction in woody tissue. However, according to the results from the water bucket experiment, the WC values given by the FDR sensor decreased by 0.974 kg m^−3^ for each increase of 1 °C.

Previous work has reported seasonal ΔStWC values within a temperate region over a range of 30 °C between summer and winter. According to our results from the water bucket experiment, an important proportion of this variation in WC found may thus be explained by temperature bias, rather than real ΔStWC, suggesting that these temperature effects have to be considered for work in regions with large ΔT (Matheny et al. 2015, Salomón et al. 2020).

Despite the small ΔT in the tropics, the effect was visible when applied to field data from the Amazon, especially data from the dry season (June–December), where ΔT is larger compared with the wet season (December–May). According to our temperature effect model, the diurnal water storage dynamics in the dry season was almost exclusively artefactual and only the wet season exhibited diurnal dynamics (Fig. 4). We quantified the effect of T and thus conclude that analyses on seasonal and diurnal water storage dynamics are potentially biased by the effect of T on the FDR signal, especially in extratropical climate regions.

## Conclusion

Our study provides a major step forward in the application of stem WC sensors in relation to key ecological and physiological questions. We demonstrate that wood density does not significantly affect the calibration. Furthermore, we show that the same calibration slope is conserved across individuals and species, including monocotyledonous palms, within and beyond the tropics. This result leads to the establishment of a general standard calibration equation for woody tissue in regards to measurements of relative WC. We show that temperature effects are non-negligible and can cause bias for analyses made at daily and seasonal scales. Our study paves the way towards a more unified approach of measuring vegetation WC, potentially finding its wider usage across tropical and other biomes.

## Supplementary Material

Supplementary_Information_24_07_tpae076

## Data Availability

All data and relevant R code scripts have been uploaded to the following GitHub repository. https://github.com/lionmartius/Martius_et_al_24_tree_water_content.

## **Statement of** inclusion

Our study brings together authors from a number of different countries, including scientists based in the country where the study was carried out. All authors were engaged early on with the research and study design to ensure that the diverse sets of perspectives they represent were considered from the onset. Whenever relevant, literature published by scientists from the region was cited.
