# Supplementary material for: Towards accurate monitoring of water content in woody tissue across tropical forests and other biomes
Source: Tree Physiol. 2024 Jul 2;44(8):tpae076. doi: 10.1093/treephys/tpae076 (PMC11299548; doi:10.1093/treephys/tpae076)
Supplement: Supplementary_Information_24_07_tpae076 [file supplementary_information_24_07_tpae076.docx]

**Supplementary Information**

1. **Resumo (português)**
2. Os ecossistemas florestais enfrentam uma exposição crescente à seca devido às alterações climáticas, necessitando de medições precisas do teor de água da vegetação (WC) para avaliar o stress da seca e os riscos de mortalidade das árvores. Embora a Reflectometria no Domínio da Frequência (FDR) ofereça um método viável para monitorizar o teor de água do caule utilizando a permissividade dieléctrica, surgem desafios devido às incertezas na calibração do sensor relacionadas com as propriedades da madeira e a variabilidade das espécies, o que impede a sua utilização mais alargada.
3. Foram amostradas árvores e palmeiras de florestas tropicais no leste da Amazônia, para avaliar como as diferenças de saída do FDR são controladas pela densidade da madeira (WD), temperatura (T) e identidade taxonómica. Três indivíduos por espécie foram abatidos e cortados em segmentos (total *n* = 262), dentro de um conjunto de dados diversificado que inclui cinco espécies de árvores dicotiledôneas e três palmeiras monocotiledôneas em uma ampla gama de densidades de madeira. O teor de água foi estimado gravimetricamente para cada segmento, utilizando uma abordagem de humedecimento/secagem temporalmente explícita, e foi examinada a relação com a saída do sensor FDR.
4. A densidade do tecido lenhoso não teve um impacto significativo na calibração, mas a identidade das espécies e a temperatura afectaram significativamente as leituras do sensor. O artefacto da temperatura foi quantitativamente importante em grandes ΔT (9.74 kg m^-3^ 10°C^-1^), o que pode ter levado a uma distorção significativa do ∆WC diário e sazonal em estudos anteriores.

Desenvolvemos o primeiro modelo linear de calibração de árvores tropicais (TTCM) com bom desempenho para valores absolutos de WC. De forma notável, demonstrámos que a sensibilidade da calibração permaneceu consistente entre as espécies, permitindo a criação de um modelo simplificado de um declive para medições precisas e independentes das espécies da WC relativa.

1. O nosso modelo de um declive serve como a primeira calibração globalmente aplicável e independente de espécies para avaliar a WC relativa em tecido lenhoso, oferecendo uma ferramenta valiosa para quantificar as respostas à seca e o stress em árvores e ecossistemas florestais.
2. **METER derived equation to calculate electric permittivity and VWC from raw values.**

$\varepsilon={(2.887 \times{10}^{-9}\times{RAW}^{3}-2.080 \times{10}^{-5}\times{RAW}^{2}+5.276 \times{10}^{-2}\times RAW-43.39)}^{2}$

$\Theta\left( \frac{m^{3}}{m^{3}} \right)=3.879\times{10}^{-4}\times RAW-0.6956$

<https://www.labcell.com/media/146679/teros%2011%20manual.pdf>

1. **Protocol for monitoring stem water content in trees**

Sensors application protocol and code for data processing has been made available within the following GitHub repository:

[*https://github.com/lionmartius/Splish-Splash-Sap*](https://github.com/lionmartius/Splish-Splash-Sap)

1. **Wood block – temperature experiment**


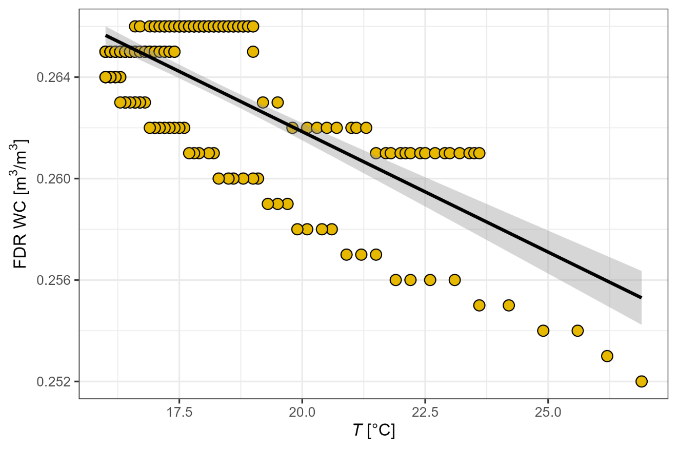


Figure 1 SI IV: While the slope coefficient for the wood block temperature experiment was highly similar to that of the water-experiment, the model performed less well and the warming-up phase (upper part of the curve) exhibits a slight shift in the intercept. This is possibly caused by humidity entering the installation holes, as the sensors were not sealed with sealants to allow re-use of the sensor in the field.

1. ***Retrieving absolute water content from the OSC***

The OSC was derived by first-order differencing, which exploits the existence of a constant slope across species and disregards the random species-specific intercept differences. Hence, the practicality of the OSC is restricted to physiological analyses based on RWC only. Yet, there is interest in working with absolute rather than relative WC to accurately model and quantify ecosystem fluxes. We propose in the following an efficient method to derive absolute WC from relative WC values using the OSC and field data. This approach should be applicable for all woody species, since the slopes derived for tropical trees here are not significantly different from the model derived for temperate trees (He et al. 2021).

Relative WC can be displayed by centring raw field data on the initial value and multiplying it by the slope coefficient. To proceed, we need to determine the absolute volumetric WC at the point in time when a tree is at 100% RWC in the field for each species. We rely on information from the literature and meteorological data, such as precipitation. The absolute WC_max_, which corresponds to 100% RWC, can be obtained by direct gravimetric measurements in the field using a wood corer, which is commonly used for sampling wood density estimations. Thus, peak values within the field dataset, typically occurring after rainy events toward the end of the rainy season, are assigned the measured absolute WC_max_. Hence, accurate and species-specific calibration models based on the constant slope and a field-derived intercept can be obtained without the need of conducting extensive time-consuming species-specific calibrations, as had been recommended in the past (Matheny et al. 2017). Any deviation from peak FDR sensor value can be subtracted from WC_max_ to give absolute WC for any given woody tissue. Hence, each intercept$(\beta_{0}$) can be estimated for each individual tree with the following equation based on $\sqrt{\varepsilon}$:

$$\beta_{0}={WC}_{max}-0.225 \sqrt{\varepsilon}_{FDR,max}$$

1. ***Linear Model comparison***

**Table 2:** Linear models for the relationship between volumetric water content, measured gravimetrically, and square root of the dielectric permittivity ($\sqrt{\varepsilon}$). While species ID is an important predictor, the majority of the variation explained by species lies within intercept rather than slope differences, despite a significant interaction term.

| Linear Model | *R^2^* | predictor | F value | *p - value* |
| --- | --- | --- | --- | --- |
| $\boldsymbol{lm(vwc\sim}\sqrt{\boldsymbol{\varepsilon}}\boldsymbol{)}$ | 0.84 | $\sqrt{\boldsymbol{\varepsilon}}$ | 1408.7 | <0.001 |
| $\boldsymbol{lm(vwc\sim}\sqrt{\boldsymbol{\varepsilon}}\boldsymbol{+species}\boldsymbol{)}$ | 0.90 | $\sqrt{\boldsymbol{\varepsilon}}$ | 2209 | <0.001 |
|  |  | $\boldsymbol{species}$ | 22 | <0.001 |
| $\boldsymbol{lm(vwc\sim}\sqrt{\boldsymbol{\varepsilon}}\boldsymbol{*species}\boldsymbol{)}$ | 0.91 | $\sqrt{\boldsymbol{\varepsilon}}$ | 2397.4 | <0.001 |
|  |  | $\boldsymbol{species}$ | 23.9 | <0.001 |
|  |  | $\sqrt{\boldsymbol{\varepsilon}}\boldsymbol{:species}$ | 4.1 | <0.001 |
